# PReS-FINAL-1001: Lymphocytes from the inflamed joint of juvenile idiopathic arthritis patients express reduced levels of cd73 and have a functional defect in adenosine production

**DOI:** 10.1186/1546-0096-11-S2-O1

**Published:** 2013-12-05

**Authors:** S Botta Gordon-Smith, S Eaton, S Ursu, H Moncrieffe, LR Wedderburn

**Affiliations:** 1 Rheumatology, University College London, Institute of Child Health, London, UK; 2 Surgery, University College London, Institute of Child Health, London, UK; 3 Rheumatology, Cincinnati Children's Hospital Medical Center, Cincinnati, USA

## Introduction

The nucleotidases CD39 and CD73 are responsible for the catabolism of pro-inflammatory ATP to AMP, and the consequent dephosphorylation of this nucleotide to regulatory adenosine. CD39 protein has previously been observed to be elevated (1) on JIA (Juvenile Idiopathic Arthritis) SFMC (synovial fluid mononuclear cells) with a correspondent increase in ATPase activity, while CD73 has been found decreased on JIA SFMC, potentially affecting the cells ability to synthesize suppressive adenosine.

## Objectives

To test the hypothesis that JIA SFMC have a reduced ability to synthesize adenosine.

## Methods

Samples (unsorted or sorted CD8+/CD19+) from 32 patients with JIA, 30 healthy adult and 5 age-matched controls were tested by flow cytometry and HPLC (high performance liquid chromatography). PBMC were stimulated with CpG or anti-CD3mAb and anti-CD28mAb. Data were compared by Mann-Whitney (2-tailed) and are expressed as medians; analysis was performed using Prism (v5.03, Graphpad).

## Results

As we previously described (2), JIA SFMC lymphocytes express decreased levels of CD73 compared to JIA patients and control PBMC, with the most marked difference on CD8+ and CD19+ cells. Both total SFMC and CD8+ SFMC showed a decreased ability to breakdown AMP. It was confirmed that B cells are the only cell type to co-express CD39 and CD73 and were therefore are able to generate adenosine from ATP. As B cells do not express the Adenosine deaminase-CD26 complex they cannot breakdown adenosine to inosine. Despite increased levels of CD39 on CD19+ SFMC, levels of coexpression of CD39 and CD73 were decreased on CD19+ SFMC (49%) as compared to B cells from JIA PBMC (64%, p = 0.0007) and PBMC from age matched (56%, p = 0.3) and adult controls (71%, *p *< 0.0001). To test whether the reduced CD73 expression found in the joint is related to the stimulatory phenotype of SFMC, this was modeled in vitro via stimulation of B cells via TLR9 or T cells via TCR stimulation: stimulation led to CD73 down-regulation and reduced AMPase activity compared to unstimulated PBMC.

## Conclusion

Decreased expression levels of CD73 on lymphocyte SFMC corresponded to decreased AMPase activity, the same situation as for stimulated PBMC, suggesting that JIA SFMC are less able to synthesize adenosine as a consequence of their activated status. These data suggest a functional defect within the joint of the production of anti-inflammatory adenosine.

## Disclosure of interest

None declared.
